# An Amperometric Immunosensor Based on Multi-Walled Carbon Nanotubes-Thionine-Chitosan Nanocomposite Film for Chlorpyrifos Detection

**DOI:** 10.3390/s121217247

**Published:** 2012-12-13

**Authors:** Xia Sun, Yaoyao Cao, Zhili Gong, Xiangyou Wang, Yan Zhang, Jinmei Gao

**Affiliations:** School of Agriculture and Food Engineering, Shandong University of Technology, No.12, Zhangzhou Road, Zibo 255049, China; E-Mails: sunxia2151@sina.com (X.S.); wenlansishu@163.com (Y.C.); gzhl1988@126.com (Z.G.); 15264312165@163.com (Y.Z.); gaojinmei9000@163.com (J.G.)

**Keywords:** multi-walled carbon nanotubes, thionine, chitosan, chlorpyrifos, immunosensor

## Abstract

In this work, a novel amperometric immunosensor based on multi-walled carbon nanotubes-thionine-chitosan (MWCNTs-THI-CHIT) nanocomposite film as electrode modified material was developed for the detection of chlorpyrifos residues. The nanocomposite film was dropped onto a glassy carbon electrode (GCE), and then the anti-chlorpyrifos monoclonal antibody was covalently immobilized onto the surface of MWCNTs-THI-CHIT/GCE using the crosslinking agent glutaraldehyde (GA). The modification procedure was characterized by using cyclic voltammetry (CV) and electrochemical impedance spectroscopy (EIS). Under the optimized conditions, a linear relationship between the relative change in peak current of different pulse voltammetry (DPV) and the logarithm of chlorpyrifos solution concentration was obtained in the range from 0.1 to 1.0 × 10^5^ ng/mL with a detection limit of 0.046 ng/mL. The proposed chlorpyrifos immunosensor exhibited high reproducibility, stability, and good selectivity and regeneration, making it a potential alternative tool for ultrasensitive detection of chlorpyrifos residues in vegetables and fruits.

## 1. Introduction

An immunosensor is a kind of biosensor that provides concentration-dependent signals by using antibodies (Ab) or antigens (Ag) as the specific sensing element [1,2]. Recently, electrochemical immunosensors have incited the interest of scholars because of their sensitivity, highly selectivity, convenience and inexpensiveness, and they have been successfully applied in environmental analysis [3], the food industry [4,5], and clinical chemistry [6,7].

In light of the potential harm resulting from human exposures to a broad range of chemical contaminants, it is necessary to develop rapid approaches for assessing internal exposure and the resulting health hazards [8]. Insecticides can be detected using UV-visible spectroscopy [9], enzyme-linked immunosorbent assay [10,11], Fourier Transform infrared spectroscopy [12], gas chromatography-mass spectroscopy [13–15], *etc*. However, these methods require expensive instrumentation, complicated pretreatment procedures and professional operators, which limits their application for real-time detection. There is an urgent need to develop a simple, rapid and cost-effective technique for the detection of desired pesticides. For these reasons, the development of rapid and efficient monitoring methods for recognitive and quantitative detection of pesticide residues in food and environment becomes more and more important.

As is well known, semiconductor multi-walled carbon nanotubes (MWCNTs), have unique electrical and mechanical properties, high surface area, and are proven to promote electron transfer between electrochemically active compounds and electrodes [16–18]. Cao *et al*. have developed an electrochemical immunosensor using poly(l-arginine)/multi-walled carbon nanotubes composite film with functionalized gold nanoparticles for the sensitive detection of casein [19]. Sun’s group have used MWCNTs for the adsorption of carcinoembryonic antibodies to construct a sensitive label-free electrochemical immunoassay [20]. Chitosan (CHIT), a derivative of the natural polysaccharide chitin, is known for its excellent film-forming and adhesion properties, together with non-toxicity and good biocompatibility, which makes it a promising matrix for biomaterial immobilization [21–23]. It is reported that CHIT-MWCNTs [24–27] has been used for fabricating various biosensors as they can provide a suitable microenvironment for immobilizing biomolecules and promoting electron transfer to enhance the sensitivity of the immunosensor. Based on this, Zhao *et al*. have developed a disposable immunosensor for the rapid detection of *Shigella flexneri*[28]. Huang *et al*. have designed a disposable electrochemical immunosensor for the detection of carcinoembryonic antigen based on Au nanoparticles/multi-walled carbon nanotubes-chitosan composite films [29]. Thionine (THI), as a perfect electronic mediator, it can also enhance electron conductivity [30–32]. Zhang *et al*. have modified a screen-printed carbon electrode using THI for the detection of *Enterobacter sakazakii*[32]. Ran *et al*. have also used THI to modify the glassy carbon electrode for detecting α-fetoprotein [33].

As described above, MWCNTs have the ability to provide high external surface area and promote electron transfer. However, untreated MWCNTs are extremely hydrophobic and tend to assemble into bundles, which make them tricky to process. Therefore, it is necessary to find effectual dispersants for MWCNTs. CHIT has excellent film forming and adhesion ability, and it can dissolve MWCNTs adequately, What’s more, due to its large amount of hydrophilic amino groups (-NH_2_), THI has a planar aromatic structure, which allows strong interaction with MWCNTs through π-π stacking force, that can further enhance the electroactivity of MWCNTs. To the best of our knowledge, there were no references to the application of MWCNTs-THI-CHIT composite film to prepare immunosensors for the detection of chlorpyrifos. In the paper we explore a novel immunosensor with the ingenious combination of MWCNTs, THI and CHIT. The immunosensor provided a simple, economic, sensitive and specific method for chlorpyrifos detection. Moreover, real sample analysis was performed to evaluate the proposed immunosensors.

## 2. Experimental

### 2.1. Apparatus

The cyclic voltammetry (CV), electrochemical impedance spectroscopy (EIS) and different pulse voltammetry (DPV) measurements were performed with a CHI 660D electrochemical workstation (Shanghai Chenhua Co., Shanghai, China). The working electrode was the glassy carbon electrode (Φ = 3 mm), a saturated calomel electrode (SCE) and a platinum electrode were used as reference and auxiliary electrodes, respectively.

### 2.2. Reagents

Anti-chlorpyrifos monoclonal antibody, chlorpyrifos, and bovine serum albumin (BSA, 96–99%) were all purchased from Sigma (St. Louis, MO, USA). Thionine was purchased from WoKai Co., Ltd. (Shanghai, China). MWCNTs were obtained from Nanotech Port Co. (Shenzhen, China). Anti-chlorpyrifos monoclonal antibody was prepared by dissolving 0.01 M phosphate buffer solution (PBS, pH 7.5). CHIT (95% deacetylation), ethanol and other chemicals were used of analytical grade.

### 2.3. Preparation of the MWCNTs-THI-CHIT Composite

MWCNTs-CHIT composite could be prepared according to the reported work [34]. First of all, CHIT solution (0.5 wt%) was prepared by dissolving 0.5 g CHIT powder in 100 mL of 1.0% (v/v) acetic acid solution and stirring for 3 h at room temperature. Then 5 mL of the above CHIT solution was mixed with 5 mg of MWCNTs, and ultrasonicated at room temperature until homogenous black suspension was obtained. Five mg THI was dispersed in 5 mL MWCNTs-CHIT solution, ultrasonicated at room temperature at least 12 h until complete dissolution, and the non-integrated thionine removed to obtain the MWCNTs-THI-CHIT composite.

### 2.4. Fabrication of the Immunosensor

The glassy carbon electrode (GCE) (Φ = 3 mm) was polished carefully with Al_2_O_3_ powder of 0.3 and 0.05 μm to a mirror-like surface, then cleaned ultra-sonically with distilled water, 6 M nitric acid, absolute ethanol and distilled water 5 min and dried with nitrogen.

**Figure 1.**
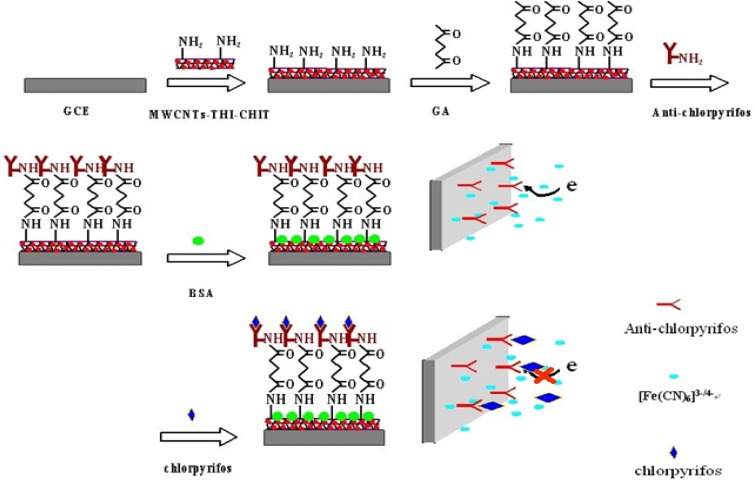
Schematic illustration of the immunosensor fabrication process.

Six μL of the prepared MWCNTs-THI-CHIT composite was dropped onto the surface of the GCE, which was then rinsed with distilled water to remove loosely adsorbed composite. To immobilize the anti-chlorpyrifos antibody onto the electrode surface, first, 3 μL of glutaraldehyde (GA) solution (2.5%) was added onto electrode surface and incubated for 30 min. After washing, 6 μL of anti-chlorpyrifos antibody solution (200 ng/mL) was added onto the electrode surface and incubated at 4 °C overnight. In order to block nonspecific binding sites, the electrode was immersed into 2.5% of BSA solution for another 1 h. The modified BSA/anti-chlorpyrifos/GA/MWCNTs-THI-CHIT/GCE immunosensor was thus obtained and stored at 4 °C when not in use. A schematic illustration of the stepwise procedures for the fabrication of the immunosensor is shown in Figure 1.

### 2.5. Electrochemical Measurements

The formation of antigen and antibody complexes was performed by immersing the immunosensor into 0.1 M PBS (pH 7.5) containing various concentrations of chlorpyrifos at 25 °C. The steps of the immunosensor fabrication procedures were investigated by CV and EIS in 0.1 M pH 7.5 PBS containing 5 mM [Fe(CN)_6_]^3−/4−^ and 0.1 M KCl at room temperature. The relative change in peak current (%ΔI) of the immunosensor before and after interaction between anti-chlorpyrifos antibody and chlorpyrifos was measured by DPV. %ΔI was calculated as follows:

$$\%\Delta \text{I}=\frac{{\text{I}}_{\text{p, BSA}}-{\text{I}}_{\text{p, chlorpyrifos}}}{{\text{I}}_{\text{p, BSA}}}\times 100\%$$

where I_p,BSA_ and I_p,chlorpyrifos_ were the peak current of the DPV before and after reaction to the antigen, respectively.

## 3. Results and Discussion

### 3.1. Characteristics of MWCNTs-CHIT Composite and MWCNTs-THI-CHIT Composite

**Figure 2.**
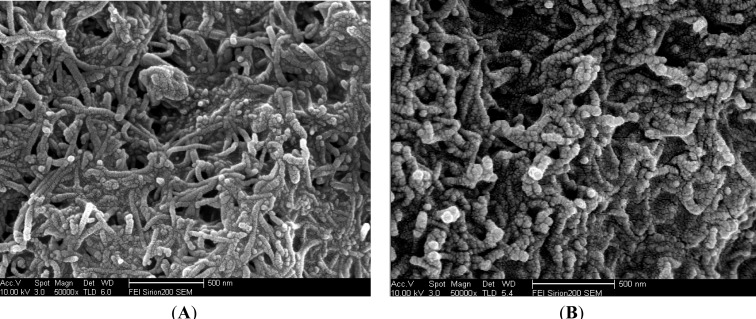
SEM images of (**A**) MWCNTs-CHIT/GCE and (**B**) MWCNTs-THI-CHIT/GCE.

Figure 2(A) shows the SEM image of the MWCNTs-CHIT modified GCE surface, where the presence of multi-walled carbon nanotubes is obvious. As shown in Figure 2, a striking difference could be discerned between the microstructures of the MWCNTs-CHIT modified with thionine (Figure 2(B)) and MWCNTs-CHIT (Figure 2(A)). The SEM image of MWCNTs-THI-CHIT film clearly shows the presence of thionine, which becomes thicker than that of MWCNTs-CHIT, indicating that thionine has conjugated with MWCNTs.

### 3.2. Electrochemical Impedance Analysis

#### 3.2.1. Cyclic Voltammetry Analysis

**Figure 3.**
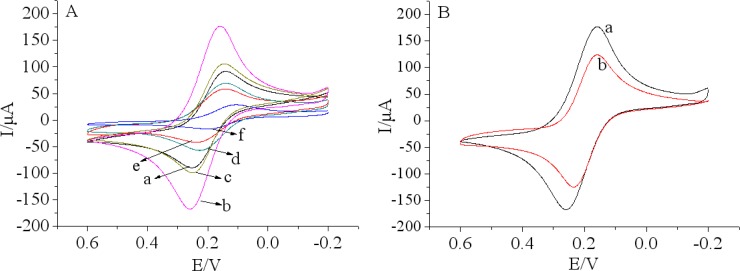
(**A**) Cyclic voltammograms: (a) bare GCE; (b) MWCNTs-THI-CHIT/GCE; (c) GA/MWCNTs-THI-CHIT/GCE; (d) anti-chlorpyrifos/GA/MWCNTs-THI-CHIT/GCE; (e) BSA/anti-chlorpyrifos/GA/MWCNTs-THI-CHIT/GCE; (f) chlorpyrifos/BSA/anti-chlor-pyrifos/GA/MWCNTs-THI-CHIT/GCE; (**B**) Cyclic voltammograms: (a) MWCNTs-THI-CHIT/GCE; (b) MWCNTs-CHIT/GCE recorded in pH 7.5 PBS containing 5.0 mM [Fe(CN)_6_]^3−/4−^ and 0.1 M KCl.

The stepwise assembly of the immunosensor was characterized by CV using 5.0 mM [Fe(CN)_6_]^3−/4−^ with 0.1 M KCl as the redox probe, and the results presented in Figure 3(A).

There was an obvious increase of the amperometric current after MWCNTs-THI-CHIT composite was coated onto the GCE (curve b) compared with the bare GCE (curve a), indicating that the use of MWCNTs significantly enhanced the electrical conductivity of the electrode [35]. At the same time, we can observe MWCNTs-THI-CHIT composite (curve a) displayed better conductivity than the MWCNTs-CHIT (curve a) composition (Figure 3(B)). As we know, THI has a planar aromatic structure and acts as a good redox probe, which was entrapped in MWCNTs, and efficiently connected with the GCE for facile charge transfer [36]. However, after GA was dropped on the MWCNTs-THI-CHIT/GCE (curve c) and Ab were immobilized on the layer (curve d), the peak current obviously decreased due to GA and Ab hindering the shuttle of electrons to the electrode surface. Subsequently, a further decrease of the peak currents might attribute to the BSA, which could block the nonspecific sites and hinder the transmission of electrons toward the electrode surface when the modified immunosensor was incubated with BSA (curve e). There was a further decrease of current response (curve f), after the immunosensor was incubated with a certain concentration of chlorpyrifos solution. This result was ascribed to the immunocomplex, which partly shielded the active center of mediator and hindered the transmission of electrons towards the electrode surface.

#### 3.2.2. Electrochemical Impedance Analysis

**Figure 4.**
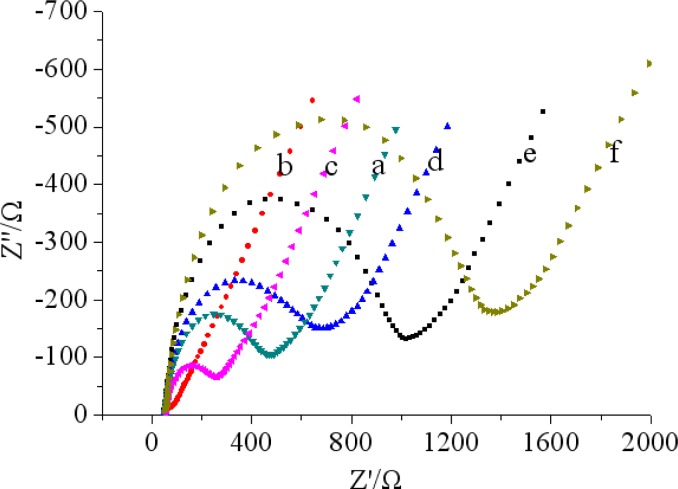
Electrochemical impedance spectroscopy: (a) bare GCE; (b) MWCNTs-THI-CHIT/GCE; (c) GA/MWCNTs-THI-CHIT/GCE; (d) anti-chlorpyrifos/GA/MWCNTs-THI-CHIT/GCE; (e) BSA/anti-chlorpyrifos/GA/MWCNTs-THI-CHIT/GCE; (f) chlorpyrifos/BSA/anti-chlorpyrifos/GA/MWCNTs-THI-CHIT/GCE recorded in pH 7.5 PBS containing 5.0 mM [Fe(CN)_6_]^3−/4−^ and 0.1 M KCl.

Electrochemical impedance spectroscopy (EIS) studies of different electrodes have been investigated in phosphate buffer (pH 7.5 and 5.0 mM [Fe(CN)_6_]^3−/4−^ with 0.1 M KCl). A Nyquist impedance spectrum generally includes a semicircular portion and a linear portion. The semicircle portion in the high frequency range corresponds to the electron-transfer-limited process and the linear portion at the low frequencies represents the diffusion-limited process. The semicircle diameter equals the electron transfer resistance (Ret), the smaller semicircular diameter indicates the faster electron transfer. As shown in Figure 4, a small semicircular diameter could be observed for bare GCE (curve a).

While MWCNTs-THI-CHIT composite was deposited on the bare GCE electrode, we found that the Nyquist diagram was almost a straight line, which demonstrated that MWCNTs-THI-CHIT film had excellent conductivity. It was clear that the semicircle diameter increased after the steps (curves c–f). The increased semicircular diameter implied a higher Ret, possibly due to an increase in the thickness and compactness. These results were corresponded well with that of CV and demonstrated the stepwise modifying process of the electrode.

### 3.3. Effect of Concentrations of THI in MWCNTs-THI-CHIT Composite

**Figure 5.**
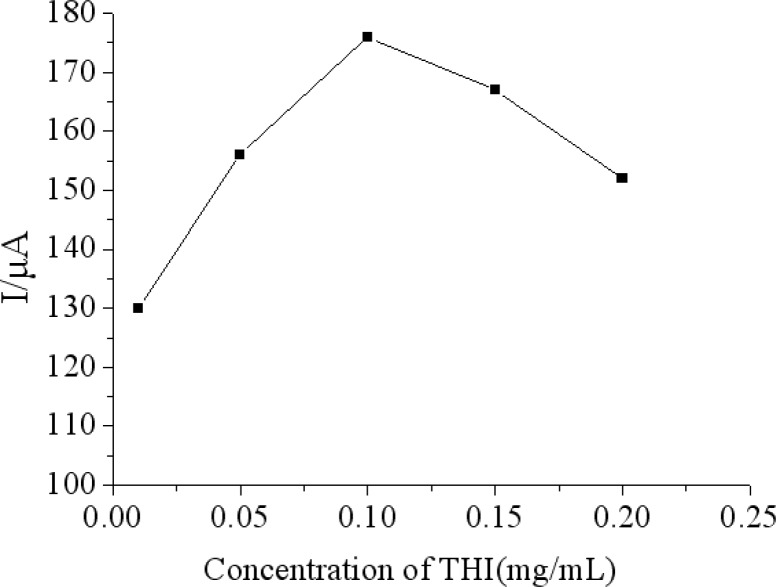
Effect of the concentrations of THI in MWCNTs-THI-CHIT composite on the immunosensor with 200 ng/mL chlorpyrifos response by the relative change in peak current of DPV (%ΔI) in 0.1 M PBS (pH 7.5) containing 5.0 mM [Fe(CN)_6_]^3−/4−^ and 0.1 M KCl.

The effect of the concentrations of THI in the MWCNTs-THI-CHIT film on the response of the immunosensor was investigated. With the increase of the GS concentration from 0.1 to 1.5 mg/mL, the current response of the electrode was first increased to the maximum at 1.5 mg/mL and then decreased (Figure 5). Since the increase of THI concentration improved, the electroactivity of MWCNTs improved, which helped to improve the sensitivity. With further increases of THI concentration, the electroactivity of MWCNTs was improved continually, but the influence of the antibody-antigen immunocomplex on the amperometric response was weakened, so 1.0 mg/mL of THI was selected as the optimal condition to prepare the immunosensor.

### 3.4. Effect of pH of the Working Buffer

**Figure 6.**
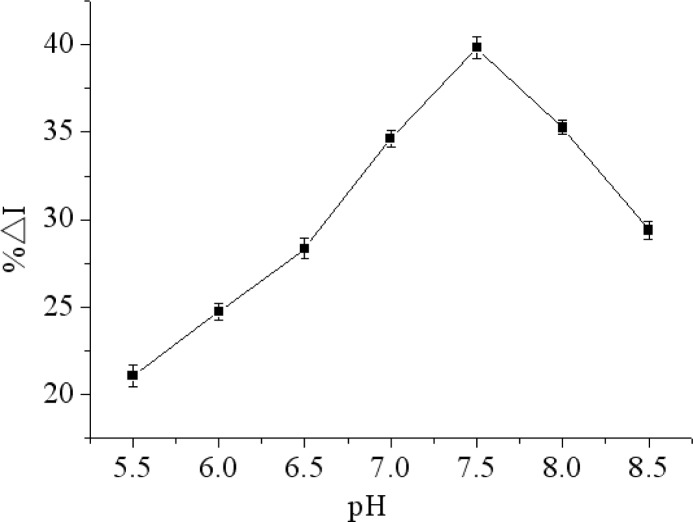
Effect of the pH of the detection solution on the immunosensor with 200 ng/mL chlorpyrifos response by the relative change in peak current of DPV (%ΔI) in 0.1M PBS (pH 7.5) containing 5.0 mM [Fe(CN)_6_]^3−/4−^ and 0.1 M KCl.

There is an obvious effect of pH on the immunosensor performance. A strongly acidic or alkaline environment can destroy the microstructure and activity of the protein. Furthermore, THI cannot perform its redox process without a proton. Therefore, pH would affect the electrochemical performance of THI. In this work, the influence of pH on the immunosensor was tested over a pH range from 5.5 to 8.5 in the presence of constant concentrations of chlorpyrifos at 4 °C. As shown in Figure 6, the maximum relative change in peak current of DPV (%ΔI) occurred at pH 7.5. In order to obtain maximum bioactivity of immunoprotein and sensitivity of immunosensor, pH 7.5 was selected through the research.

### 3.5. Effect of Incubating Time

The incubation time is a major factor of forming immunocomplex on electrode surface. The effect of incubation temperature on the maximum relative change in peak current of DPV (%ΔI) was studied in the incubation time range from 5 to 35 min.

**Figure 7.**
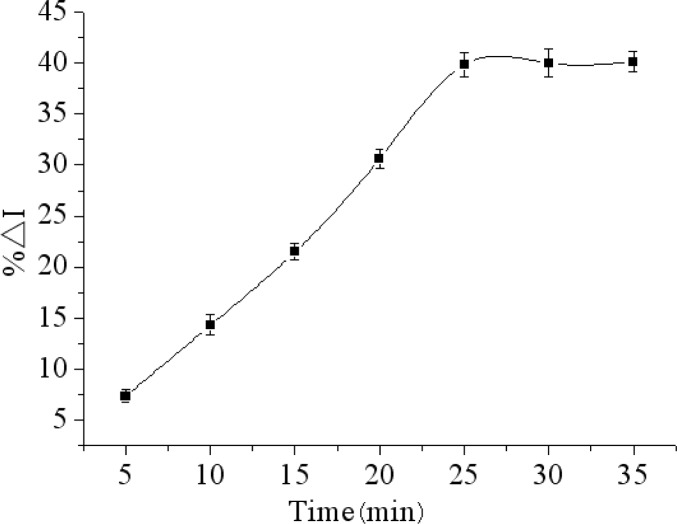
Effect of incubation time on the immunosensor inubated with 200 ng/mL chlorpyrifos response by the relative change in peak current of DPV (%ΔI) in 0.1M PBS (pH 7.5) containing 5.0 mM [Fe(CN)_6_]^3−/4−^ and 0.1 M KCl.

As the results show in Figure 7, the %ΔI increased with incubation time increasing, and then reached a constant value when the incubation time was longer than 25 min. Thus, the incubation time of 25 min was selected as the optimal incubation conditions.

### 3.6. Determination of Chlorpyrifos

**Figure 8.**
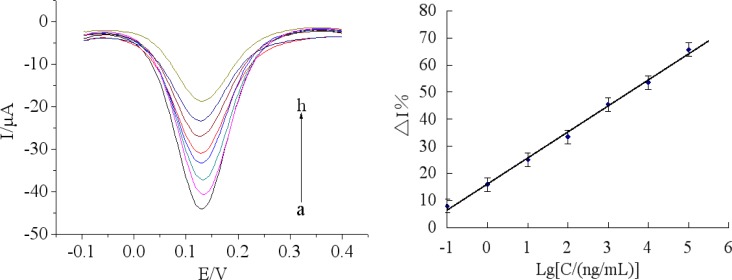
(**A**) The DPVs after the immunosensors incubated in different concentrations of chlorpyrifos standard sample solution (from a to h):0, 0.1, 1.0, 10.0, 1.0 × 10^2^, 1.0 × 10^3^, 1.0 × 10^4^ and 1.0 × 10^5^ ng/mL under the optimal conditions; (**B**) The calibration plots of the relative change in peak current of DPV (%ΔI) of the proposed immunosensor *versus* the logarithm of chlorpyrifos concentration under optimal conditions. The amperometric detection was performed 0.1M PBS (pH 7.5) containing 5.0 mM [Fe(CN)_6_]^3−/4−^ and 0.1 M KCl.

Under optimal conditions, the immunosensor was incubated in a series of different concentrations of chlorpyrifos solutions. Figure 8(A) showed the BSA/anti-chlorpyrifos/GA/MWCNTs-THI-CHIT/GCE sensors immersed in various concentrations of chlorpyrifos. The calibration plots between %ΔI and different concentrations of chlorpyrifos were shown in Figure 8(B), which shows the linear curve ranging from 0.1 ng/mL to 1.0 × 10^5^ ng/mL with a regression equation: y = 9.5771 lgC (ng/mL) + 16.220 (R^2^ = 0.9964). The detection limit was 0.046 ng/mL, which was calculated at a signal to noise ratio of 3 (S/N = 3). As shown in Table 1, compared with other reported methods, the immunosensor had a good detection limit, indicating that the proposed BSA/anti-chlorpyrifos/GA/MWCNTs-THI-CHIT/GCE sensor is reliable for the determination of chlorpyrifos pesticides.The performance of the BSA/anti-chlorpyrifos/GA/MWCNTs-THI-CHIT/GCE sensor was compared with other reported immunosensors for the detection of chlorpyrifos previously. As shown in Table 1, compared with other methods, the immunosensor has a relative large linear range and lower detection limit.

### 3.7. Reproducibility, Stability, Selectivity and Regeneration of the Immunosensor

The reproducibility of the immunosensor was examined by determining 200 ng/mL chlorpyrifos levels for five replicate measurements. The intra-assay and inter-assay coefficients of variation with the above method were 4.3% and 5.7%, indicating satisfactory reproducibility of the proposed immunosensor. The stability of the modified electrode was evaluated by measuring the current response. The prepared immunosensors were suspended over the PBS (pH 7.5) at 4 °C when not use. After 24 days of storage, no apparent change for the detection of the same chlorpyrifos concentration was found, which retained 90.5% of the initial response. These observations could be attributed to the fact that the antibody was attached firmly to the MWCNTs-THI-CHIT composites and the stability of the immunosensor was quite good.

**Figure 9.**
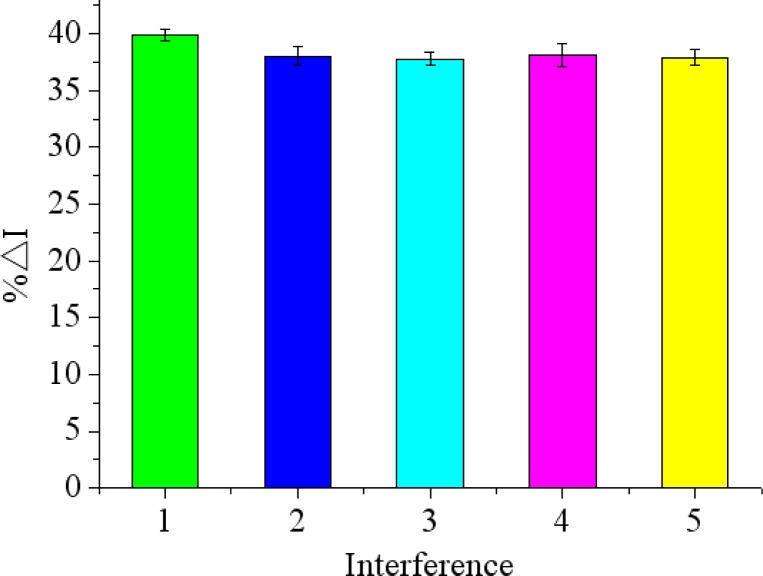
The relative change in peak current (%ΔI) of proposed immunosensor to: (**1**) 200 ng/mL chlorpyrifos, (**2**) 200 ng/mL chlorpyrifos + 200 ng/mL carbofuran, (**3**) 200 ng/mL chlorpyrifos + 200 ng/mL phoxim, (**4**) 200 ng/mL chlorpyrifos + 200 ng/mL carbaryl, (**5**) 200 ng/mL chlorpyrifos + 200 ng/mL 3-hydroxycarbofuran.

To examine the selectivity of the immunosensor for chlorpyrifos detection, other non-target small molecule pesticides such as carbofuran, phoxim, carbaryl, 3-hydroxycarbofuran were chosen in the experiment. As shown in Figure 9, there was just a difference of less than 3.8% in peak current (%ΔI) between the peak current responses in the chlorpyrifos solutions with and without interference. It indicated that the proposed immunosensor has a high degree of selectivity for chlorpyrifos detection.

**Figure 10.**
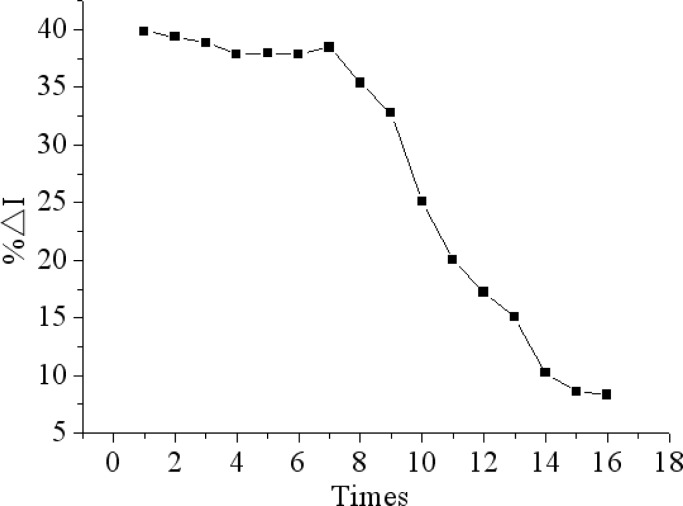
The effect of number of regeneration cycles on the immunosensor response.

The proposed immunosensor can be reactivated by immersion in 0.1M glycine-HCl solution (pH 2.8) for 5 min to remove the immunocomplex for detecting chlorpyrifos again. The antibody-antigen complex can be dissociated by lowering the pH value of reaction solution. When the antibody-antigen complex was dissociated, and the steric hindrance was minimized, consequently, the response of the immmunosensor was resumed. Figure 10 shows that no remarkable decrease in current response was observed after seven regeneration cycles. However, with the further increase of regeneration times, the current response began to obviously decrease. The reason may be that anti-chlorpyrifos antibody can gradually shell off during continuous processing by a glycine-HCl buffer and even denature [42].

### 3.8. Analysis of Real Samples

In order to evaluate the feasibility of the proposed immunosensor for real sample analysis, cabbage, lettuce and Chinese chives samples were cleaned three times using double-distilled water and were spiked with chlorpyrifos solutions of different concentration. After 24 h, samples weighing 10 g were chopped and meshed. Then the samples were extracted with 10 mL mixed solution of acetone/0.1 M pH 7.5 PBS (1/9, v/v) by shaking for 45 min. Then the extract was separated from the insoluble materials by centrifigation for 10 min at 10,000 rpm and the supernatants were directly detected by DPV without extraction or preconcentration.

As shown in Table 2, the recovery was in the range of 85.2%–104.3%, furthermore, the recovery of different vegetable samples was different, indicating real samples have an influence on the immunosensor detection results.

## 4. Conclusions

In summary, we have introduced a strategy for preparing a label-free amperometric MWCNTs-THI-CHIT sensor, in which anti-chlorpyrifos monoclonal antibodies were successfully immobilized on the GCE surface for the detection of chlorpyrifos. The high stability of the MWCNTs-THI-CHIT-modified glassy carbon electrode makes it easier to immobilize antibodies on the proposed immunosensor and ensure the efficient activity retention of loaded immunoreactants, which can improve the sensitivity and linear range of the immunosensor. The immunosensors can be easily regenerated and reused due to the introduction of CHIT and THI, which can protonate -NH_2_ groups. The constructed immunosensor prossessed prominent characteristic and performance features such as a wider linear range, better reproducibility, specificity, acceptable stability, and low detection limit, and has potential application for the detection of other pesticides or compounds. The developed biosensor provides a new promising tool for chlorpyrifos analysis.

## Figures and Tables

**Table 1.** Comparison with other reported immunosensors for the detection of chlorpyrifos.

**Electrode**	**Liner range (ng/mL)**	**Detection limit (ng/mL)**	**References**
AChE/CPBA/GR-AuNPs/GCE	0.5–10	0.1	[37]
	10–100		
SPR	0.02–200	0.05	[38]
AChE/[BMIM][BF_4_]/MWCNT/CP	3.5–350	1.4	[39]
dsCT-DNA/PANI-PVS/ITO	0.5–200	0.5	[40]
AChE/MWCNTs-TCNQ/SPE	0.35–35	0.1	[41]
BSA/anti-chlorpyrifos/GA/MWCNTs-THI-CHIT/GCE	0.1–1.0 × 10^5^	0.046	This work

**Table 2.** The recovery of chlorpyrifos in real samples.

**Sample**	**Taken (ng/mL)**	**Found (ng/mL)**	**Recovery (%)**	**RSD (%) (n = 3)**
Cabbage	10	10.37	103.7	4.76
	100	89	89.0	4.58
	1,000	978	97.8	4.95
Lettuce	10	10.43	104.3	3.98
	100	95.3	95.3	4.16
	1,000	895	89.5	4.52
Chinese chives	10	10.28	102.8	4.72
	100	85.2	85.2	5.12
	1,000	870	87.0	4.96
